# Genomic Analysis of *Cronobacter condimenti* s37: Identification of Resistance and Virulence Genes and Comparison with Other *Cronobacter* and Closely Related Species

**DOI:** 10.3390/ijms25168622

**Published:** 2024-08-07

**Authors:** Anna Berthold-Pluta, Ilona Stefańska, Stephen Forsythe, Tamara Aleksandrzak-Piekarczyk, Lidia Stasiak-Różańska, Monika Garbowska

**Affiliations:** 1 Department of Technology and Food Assessment, Division of Milk Technology, Institute of Food Sciences, Warsaw University of Life Sciences—SGGW, 02-787 Warsaw, Poland; anna_berthold@sggw.edu.pl (A.B.-P.); lidia_stasiak_rozanska@sggw.edu.pl (L.S.-R.); 2 Department of Preclinical Sciences, Institute of Veterinary Medicine, Warsaw University of Life Sciences—SGGW, 02-787 Warsaw, Poland; ilona_stefanska@sggw.edu.pl; 3 FoodMicrobe.com Ltd., Keyworth, Nottinghamshire NG12 5GY, UK; sforsythe4j@gmail.com; 4 Institute of Biochemistry and Biophysics, Polish Academy of Sciences (IBB PAS), 02-106 Warsaw, Poland; tamara@ibb.waw.pl

**Keywords:** *Cronobacter*, *Franconibacter*, *Siccibacter*, cgMLST analysis, virulence factors, antimicrobial resistance

## Abstract

*Cronobacter condimenti* are environmental commensals that have not been associated with any clinical infections. To date, they are the least understood and described *Cronobacter* species within the genus. The objective of this study was to use a draft genome sequence (DGS) of the *Cronobacter condimenti* strain s37 to screen for genes encoding for antibiotic resistance, virulence, response to environmental stress, and biofilm formation. The strain was isolated in Poland from commercial small radish sprouts. This is the second genome of this species available in the GenBank database. The comparative genome analysis (cgMLST) of *C. condimenti* s37 with other *Cronobacter* spp. including the pathogenic species *C. sakazakii* and the plant-associated closely related genera *Franconibacter* and *Siccibacter* was also performed. The assembled and annotated genome of the *C. condimenti* s37 genome was 4,590,991 bp in length, with a total gene number of 4384, and a GC content of 55.7%. The s 37 genome encoded for genes associated with resistance to stressful environmental conditions (metal resistance genes: zinc, copper, osmotic regulation, and desiccation stress), 17 antimicrobial resistance genes encoding resistance to various classes of antibiotics and 50 genes encoding for the virulence factors. The latter were mainly genes associated with adhesion, chemotaxis, hemolysis, and biofilm formation. Cg-MLST analysis (3991 genes) revealed a greater similarity of *C. condimenti* s37 to *S. turicensis*, *F. pulveris*, and *C. dublinensis* than to other species of the genus *Cronobacter*. Studies on the diversity, pathogenicity, and virulence of *Cronobacter* species isolated from different sources are still insufficient and should certainly be continued. Especially the analysis of rare strains such as s37 is very important because it provides new information on the evolution of these bacteria. Comparative cgMLST analysis of s37 with other *Cronobacter* species, as well as closely related genera *Franconibacter* and *Siccibacter*, complements the knowledge on their adaptability to specific environments such as desiccation.

## 1. Introduction

The genus *Cronobacter* spp. belongs to the family *Enterobacteriaceae* and currently includes seven species, such as *C. sakazakii*, *C. malonaticus*, *C. turicensis*, *C. muytjensii*, *C. dublinensis*, *C. condimenti*, and *C. universalis*. *Cronobacter* species can cause serious illness (mortality rates ranging from 40% to 80%) in newborns and infants, including necrotizing enterocolitis, sepsis, and meningitis. Many of these infections are caused by consuming contaminated powdered infant formula (PIF) [1]. In 2022, in the United States, *Cronobacter* was associated with infections in infants, including two deaths, after consuming PIF [2]. These pathogens also cause diseases in adults, such as wound and urinary tract infections, sepsis, vaginitis, pneumonia, and foodborne gastroenteritis, but clinical symptoms in adults are milder than in newborns [3,4]. Infections caused by *Cronobacter* spp. are rare [2]. Generally, these bacteria exhibit several typically pathogenic features, such as the ability to invade human intestinal cells and cross the blood–brain barrier, multiply in macrophages, and evade the host immune response [5].

*Cronobacter condimenti* was first isolated in Slovakia and the type strain is 1330^T^ (CECT 7863^T^, LMG 26250^T^) [6]. *C. condimenti* are environmental commensals that have not been associated with any clinical infections. Due to the rarity of their isolation, they are the least understood and described *Cronobacter* species within the genus to date. The majority of *Cronobacter* isolates from clinical sources causing serious disease are *C. sakazakii* and *C. malonaticus*, with *C. turicensis*, *C. universalis*, *C. dublinensis*, and *C. muytjensii* being less frequently reported. Bacteria of the genus *Cronobacter*, although initially associated only with neonatal infections, are now recognized as causing infections mainly in adults [7,8,9].

The first isolate of *C. condimenti* (1330^T^) was from spiced sausages. Given that members of the *Cronobacter* genus are not frequently isolated from meat products, it was probable but unproven that *C. condimenti* was from the spice ingredient of the sausages. The phenotypic characteristics of the reference strain *C. condimenti* 1330^T^ have shown that, unlike other *Cronobacter*, this species is immobile, produces indole from tryptophan, and has more limited sources of carbon utilization compared to other *Cronobacter* species (e.g., they do not use inositol, putrescine, maltitol, or palatinose) [6]. 

The most closely related genera to *Cronobacter* are *Franconibacter* and *Siccibacter*. Strains within these latter genera were previously regarded as members of the *Cronobacter* genus. However, Stephan et al. [10] based on phylogenetic evidence and genome analysis proposed the genus *Franconibacter*, which currently includes *F. pulveris*, *F. helveticus* (previously *C. pulveris* and *C. helveticus*, respectively), and *F. daqui* (the reference strain was isolated in 2017 from Daqu by Gao et al. [11] and the genus *Siccibacter*, which includes *S. turicensis* (formerly *C. zurichensis*) and, since 2015, *S. colletis* [12]). One of the main reasons for their initial inclusion in the *Cronobacter* genus is that they have similar biochemical properties (for example, α-glucosidase activity, which is used as a marker in some diagnostic media for *Cronobacter*) [13]. The Rapid ID32E and Vitek 2 GN systems are based on biochemical identification and misidentify strains of the genera *Franconibacter* and *Siccibacter* as *Cronobacter* spp. [14]. Currently, the only means of correctly identifying strains of the genera *Franconibacter* and *Siccibacter* is DNA sequence analysis, particularly using the multilocus sequence typing (MLST) scheme developed for *Cronobacter* species [7]. This approach analyzes seven housekeeping genes, including the *fusA* gene encoding elongation factor G. The phylogenetic analysis of *fusA* is considered to best reflect the whole-genome phylogeny of *Cronobacter* and related genera. However, the MLST database reveals that *Cronobacter* and *Franconibacter* share alleles in this gene, invalidating identification based on single-locus sequencing.

*Franconibacter* and *Siccibacter* strains have been isolated mainly from fruit powders, spices, and herbs and less often from powdered infant formula (PIF). To date, there has only been one clinical report of infection from *Siccibacter* and none from *Franconibacter* [15]. Due to their close relatedness, *Franconibacter* (39 strains) and *Siccibacter* (19 strains) are included in the PubMLST *Cronobacter* database.

The genus *Cronobacter* has undergone a significant transformation during evolution, with species such as *C. sakazakii* and *C. malonaticus* exhibiting more pathogenic characteristics, while others have less impact on human health. Information on the diversity, pathogenicity, and virulence of *Cronobacter* spp. isolated from different sources is still insufficient and is currently being studied. Our current knowledge on *Cronobacter* infections includes various virulence factors, such as adherence and invasiveness in cell lines, sialic acid utilization, capsule and endotoxin production, genes encoding iron uptake systems, fimbriae, flagella, invasion, outer membranes, and serum resistance [3]. Furthermore, the literature data indicate that *Cronobacter* bacteria exhibit resistance to various antibiotics [1,4], and the presence of antibiotic resistance genes, plasmids, and mobile genetic elements have also been reported [16].

The objective of this study was to use a draft genome sequence (DFG) for the detection of genes that confer resistance to antibiotics (ARGs), virulence (VGs), response to environmental stress, and biofilm formation in *C. condimenti* s37 that had been isolated from small radish sprouts [17], which allows for more precise molecular epidemiological studies. To the best of our knowledge, *C. condimenti* s37 is the second genome sequenced strain and the fourth documented isolate of this species. Studies on the diversity, pathogenicity and virulence of *Cronobacter* spp. isolated from different sources are still insufficient and should certainly be continued. Especially the analysis of rare strains such as s37 is very important because it provides new information on the evolution of these bacteria. In addition, the comparative genome analysis (cgMLST) of strain s37 with other *Cronobacter* spp. as well as with the species of *Franconibacter* and *Siccibacter*, available in the PubMLST database, was also performed, to obtain a broader view of the evolution of the strain isolated from the commercial sprouts. Comparison with closely related genera such as *Franconibacter* and *Siccibacter* can provide information concerning the ecology of *Cronobacter* species as well as why these bacteria are able to survive under stressful environmental conditions.

## 2. Results and Discussion

### 2.1. General Features of C. condimenti s37 Genome

The genome of *C. condimenti* s37 was assembled and annotated, revealing a total size of 4,590,991 bp. It contains 4200 protein-coding genes, 88 pseudogenes, 96 RNAs, and has a GC content of 55.7%. A total of 444,972 high-quality paired-end reads were generated, leading to an assembly with 48 contigs and 43 scaffolds with an average final sequence coverage of 30x. The N50 lengths of the contigs and scaffolds were 353,823 kb and 532,226 kb, respectively. The longest scaffold was 956,796 bp, while the shortest was 288 bp.

**Figure 1:**
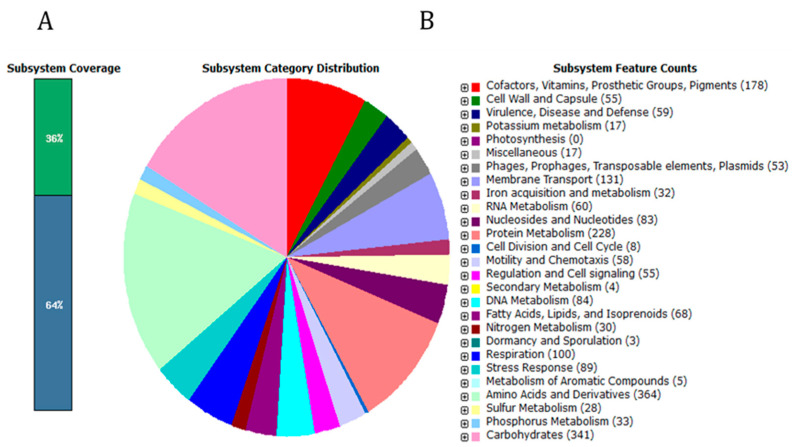
Subsystem category distribution of *C. condimenti* s37. (**A**)—the percentage of subsystem coverage (green bar corresponds to the percentage of proteins involved). (**B**)—the fraction and count (parenthesis in legend) of each subsystem feature (functional traits identified by the RAST server).

The draft genome was evaluated using the BUSCO tool with the bacteria_odb10 and enterobacterales_odb10 databases. The completeness scores were high: 97.6% for bacteria_odb10 and 99.1% for enterobacterales_odb10 (Figure S1). The genomic characteristics of the s37 strain are collated in Table 1, while the functional traits, as identified by the RAST server, are shown in Figure 1.

The coding sequences of *C. condimenti* s37 include genes involved in resistance to heavy metals such as cobalt, zinc, and cadmium, as well as genes related to copper resistance and potassium homeostasis. Additionally, genes for responding to osmotic and oxidative stress, several phage-associated traits, and antibiotic resistance were identified.

Among the genes that make up the *C. condimenti* s37 genome, there were those responsible for typical *Cronobacter* phenotypic features related to the reduction of nitrate, utilization of D-mannose, L-arabinose, D-xylose, D-mannitol, sucrose, and cellobiose. There was also the presence of five from eight of the genetic determinants of the biochemical characteristics used previously for *Cronobacter* biotyping, namely, indole (tryptophanase), malonate, myo-inositol, utilization of 4-aminobutyrate, and production of α-methyl glucoside, and the absence of three: utilization of dulcitol, palatinose, and putrescine. Hamby et al. [18], using a limited range of strains, proposed the ability to ferment inositol as a feature distinguishing pathogenic *Cronobacter* strains from non-pathogenic strains. This association is doubtful given that the inositol monophosphatase gene is found in the *C. condimenti* s37 as well as *C. universalis* and *C. dublinensis*, previously reported by Grim et al. [19].

### 2.2. Genome Comparison of C. condiment s37 with Other Cronobacter and Closely Related Species

The genome of *C. condimenti* s37 was compared with that of *C. condimenti* LMG 26250^T^ and other representatives of the genus *Cronobacter*, as well as *Franconibacter* and *Siccibacter*. These representatives included *C. sakazakii* ATCC BAA-894, *C. malonaticus* LMG 23826^T^, *C. turicensis* LMG23827^T^, *C. muytjensii* CIP 103581, *C. dublinensis* LMG 23823^T^, *C. universalis* NCTC 9529^T^, *F. helveticus* LMG 23733, *F. pulveris* Fp-G-601/05^T^, *F. daqui* LMG 29914^T^, *S. colletis* NCTC 14934^T^, and *S. turicensis* LMG 23730^T^ strains. The aim was to use strains representing the diversity across the three genera in order to identify genes of phenotypic and pathogenic interest, as well as those unique to the *C. condimenti* s37 strain.

The genome of *C. condimentii* s37 was found to contain 3991 loci, of which 44.8% (1788) were common to all 13 compared genomes (Table S1). The core genome in *C. condimenti* s37 accounted for 2216 (55.5%) core loci, of which 1788 were present in all strains included in cgMLST analysis. Because the draft genome can have sequence gaps, some genes may have incomplete fragments or were unannotated. This may result in some underestimation of the size of the core genome. The remaining 1775 (44.5%) loci in s37 strain genome were auxiliary or accessory genes, which were found in at least one of the compared genomes of other strains of genus *Cronobacter* and closely related species. 

Paralogous loci detected in cgMLST analysis are presented in Table 2, indicating the loci with multiple hits in every isolate (except those where the gene was absent). Among these genes were genes unique to *C. condimenti* (s37, LMG 26250^T^) and *C. dublinensis* 1210, including those encoding proteins for the contact-dependent growth inhibition system (CDI). CDIs are responsible for the direct physical contact of one bacterial cell with other cells nearby by delivering toxins via a receptor. These systems have been found in many microorganisms, including clinically important human pathogens. The main purpose of these systems in bacteria is to provide them with a competitive advantage for population growth under certain environmental conditions [20]. Furthermore, the closely related *Franconibacter* species lacked the PTS sugar transporter subunit IIC gene, found in *C. condimenti* s37 as well as in other *Cronobacter* and *Siccibacter* genera.

cgMLST analysis revealed that a large number (3780 out of 3991/94.7%) of loci in the s37 strain had identical alleles to those previously described in *C. condimenti* LMG26250^T^. The strain s37 unlike the reference strain *C. condimenti* LMG26250^T^ is mobile. Both strains possess the same flagellar genes; however, different alleles were found in the genes encoding the fimbria/pilus outer membrane usher protein (AFK62_RS06490|AFK62_06490) and the flagellar basal-body MS-ring/collar protein FliF (AFK62_RS12145|fliF|AFK62_12150). The bacterial flagellum is a complex macromolecular structure that drives bacterial motility and plays various roles in virulence, including assisting in colonization and invasion or biofilm formation. FliF is an inner-membrane protein that forms two ring-shaped structures (MS rings) required for flagellum assembly [21]. Furthermore, different alleles were observed in the loci two-component system sensor histidine kinase RcsC (AFK62_RS13235|rcsC|AFK62_13245). This kinase is a component of the Rcs signaling pathway, which controls various physiological functions, including envelope synthesis, cell division, and motility in prokaryotes. The Rcs regulatory cascade, which involves a multistep process between the two membrane-bound hybrid sensor kinases RcsC and RcsD and the global regulator RcsB, is one of the most complex regulatory systems in bacteria to date [22]. Moreover, *C. condimenti* s37 had proteins related to the type VI secretion system (T6SS) that differed from those identified in *C. condimenti* LMG26250T. These proteins include type VI secretion system tip protein TssI/VgrG (AFK62_RS00520|tssI|AFK62_00520, AFK62_RS00555|tssI|AFK62_00555, AFK62_RS00670|tssI| AFK62_00670), type VI secretion system protein TssA (AFK62_RS07290|tssA|AFK62_07290), and type VI secretion system ATPase TssH (AFK62_RS14105|tssH|AFK62_14125). The T6SS system was originally discovered in plant bacteria (*Rhizobium leguminosarum*) and has diverse functions in bacteria, including antibacterial and anti-host activities, mediating the uptake of metal ions, resistance to environmental stress, inhibition of bacterial infections in the host, and regulation of bacterial biofilm formation [23]. In the case of s37, rearrangement hot spot (Rhs) proteins differed from those found in the *C. condimenti* LMG26250^T^ genome. These included RHS repeat-associated core domain-containing proteins (AFK62_RS00575|AFK62_00575 and AFK62_RS00680|AFK62_00680), as well as RHS domain-containing proteins (AFK62_RS00690|AFK62_00690, AFK62_RS00700|AFK62_00700, and AFK62_RS22660|AFK62_00710). Rhs proteins are widely distributed in bacteria and eukaryotes, but their functions remain poorly understood. In enterobacteria, Rhs proteins have been shown to consist of four distinct regions, and the core may vary among family members [24].

**Figure 2:**
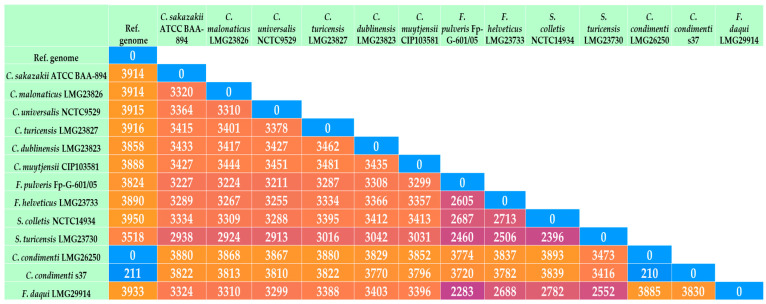
Distance matrix of *Cronobacter* species and closely related organisms (distances between taxa are calculated as the number of loci with different allele sequences).

**Figure 3:**
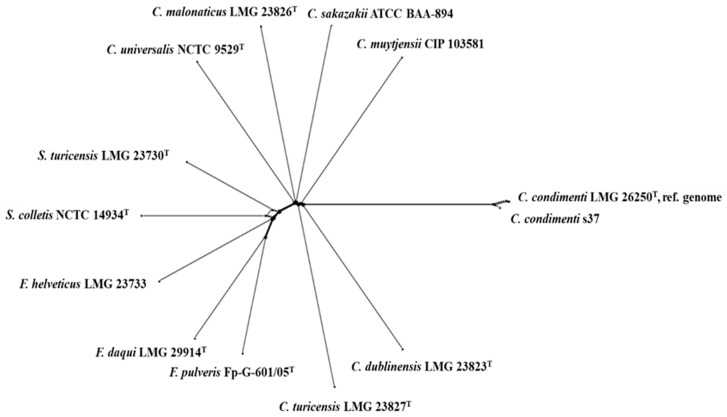
cgMLST analysis of *Cronobacter* species and closely related organisms (Splits Tree).

Interestingly, some alleles of *C. condimenti* s37 were more similar to those of strains belonging to other closely related genera (*Franconibacter* and *Siccibacter*) (Figure 2, Table S1). The similarity of *Cronobacter* with *Franconibacter* and *Siccibacter* in terms of phenotypic characteristics has been demonstrated in previous studies [13,14]. In our study, we found a relatively high similarity of *C. condimenti* s37 to *S. turicensis* LMG23730T, *F. pulveris* Fp-G-601/05T, and *C. dublinensis* LMG23823T based on allele sequence comparison and cg-MLST analysis (Figure 2 and Figure 3).

### 2.3. Genes Involved in Resistance to Environmental Stress

A number of key stress response genes were investigated. Bacteria adapt to heavy metal stress by developing various resistance mechanisms via intracellular metal-binding proteins. The sequence of *C. condimenti* s37 was found to contain several genes (*zntB*, *zupT*, *znuA*, *znuC*, and *znuB*) encoding for zinc transporter ZntB, zinc transporter ZupT, zinc ABC transporter substrate-binding protein ZnuA, zinc ABC transporter ATP-binding protein ZnuC, and Zinc ABC transporter permease subunit ZnuB. These genes are involved in zinc transport to the cytosol and outside the cytoplasm [25]. Zn is known to be a common toxic metal that co-selects for antibiotic resistance, especially when genes associated with resistance are clustered on the same genetic element [25].

In addition to the zinc resistance genes, the s37 strain genome encoded for copper homeostasis and transport, i.e., *cueO* (multicopper oxidase CueO), *copA* (copper-exporting P-type ATPase CopA), *pcoA* (multicopper oxidase PcoA), and *pcoR* (copper response-regulating transcription factor PcoR). The Cue transport system is composed of a gene encoding for periplasmic protein CopA exhibiting laccase (EC. 1.10.3.2) activity and the CueO protein which is responsible for cell periplasm protection against copper-induced damages and the reduction of Cu(I) toxicity by transporting it to the periplasm and oxidizing to less toxic Cu(II) [26]. The *pco* encodes for resistance to copper and contains seven genes, *pcoABCDRSE*. PcoA, found in the analyzed s37 strain, belongs to the family of multicopper oxidases and is deemed to be a central protein of the Pco system. 

The exceptional resistance of *C. sakazakii* to very low water activity, low pH, high temperature, and the presence of osmotically active factors in the environment has been extensively described in the literature [27,28]. The genomic sequence of *C. condimenti* s37 was found to contain various stress-response genes associated with resistance to these environmental conditions. 

The study showed that the s37 strain encodes for various factors responsible for osmotic regulation, i.e., osmotically inducible protein (OsmY), aquaporin Z, hyperosmotic potassium uptake protein (TrkH) and potassium uptake protein (TrkA and TrkG), transporters ProP and ProuU (ProVWX), glutathione-regulated potassium-efflux system protein (KefBCFG), as well as choline dehydrogenase and betaine-aldehyde dehydrogenase (BetA and BetB). In addition, it encoded genes for heat/cold shock stress factors, such as suppressor proteins (DnaJ and DnaK) and heat shock proteins (YciA, YciH, and GrpE).

The primary response of *C. sakazakii* to low water activity conditions consists in the rapid accumulation of potassium glutamate in cells, which increases internal osmotic pressure and is expected to counteract high external osmotic pressure of the low-moisture environment [27]. Genes encoding for the proteins associated with osmotic response, such as Aquaporin Z, DnaJ, and ProQ, were identified in *C. sakazakii* strains derived from dried food products, like, e.g., seasonings [8] and milk powder [16]. The secondary response of *C. sakazakii* to osmotic stress consists in the substitution of potassium glutamate with osmoprotective agents being safer to a cell, like, e.g., proline, glycine betaine, carnitine, and trehalose [27]. In the case of *E. coli*, the capture of these agents is facilitated by transporters ProP and ProU [29], which have broad substrate specificities, transporting proline, glycine betaine, and related compounds [27]. Sequence data obtained in our study demonstrated that the s37 strain possessed the *proP* gene and also the second major osmoprotective system, i.e., *ProU*, consisting of two membrane-bound proteins (ProV and ProW) and a protein bound with periplasm (ProX), coded by the *proVWX* operon. The *proP* gene and the *proVWX* operon were shown to be highly upregulated in *C. sakazakii* strains exposed to drying [27,29]. 

Choline is one of the important solutes involved in the cellular response to adverse environmental conditions. The BetA and BetB proteins are involved in the conversion of choline to betaine; first, with the participation of choline dehydrogenase (BetA), choline is converted to betaine aldehyde, and then, betaine aldehyde is converted to betaine, which is catalyzed by betaine aldehyde dehydrogenase (BetB). Both *betA* and *betB* genes were identified in the s37 strain. Previously, these genes were identified in *C. sakazakii* by Aly et al. [16], Du et al. [27], and Hu et al. [30].

Glutathione-gated potassium-efflux systems (Kef) can control K+ efflux and prevent the prolonged exposure of a cell to excess potassium ions. In this study, *kefBCFG* genes were identified in the s37 strain. The genes of this system were previously detected in *C. sakazakii* strains isolated from milk powder and salad mix [16]. As Du et al. [27] showed, the *kefB* expression was up to 12-fold higher in the strain with strong desiccation tolerance. These findings indicate that this regulatory system may be more developed in the genus *Cronobacter*, accounting for its characteristic high resistance to drying. Analyses conducted in the present study identified also two constitutive low-affinity K+ transport systems: TrkH (an integral membrane protein), *trkA*, and *trkE* encoding regulatory proteins, and a Kdp-inducible high-affinity P-type ATPase system which has a high specificity for K+ (*kdpABCDE*) in the *C. condimenti* s37 strain. Aly et al. [16] and Srikumar et al. [29] reported similar findings regarding *C. sakazakii* strains. 

In early studies on the genus *Cronobacter*, Iversen and Forsythe [31] hypothesized that this group of microorganisms were commensals associated with the plant environment. Supporting this hypothesis, several plant ecosystem-related genomic features were detected, including the metabolism of plant-associated sugars. In the *C. condimenti* s37 genome, the maltose transporter operon (*malGFEI* and *malKMT*), several α- and β-glucosidases, and genes for the transport and utilization of xylose (xylose ABC transporter), which is a precursor of hemicellulose, and for oligogalacturonate breakdown, the principal component of pectin, were found. The genes (*xylA*) indicating the capability for xylose degradation were earlier reported by Chase et al. [32] and Jang et al. [33] for *C. sakazakii* strains and by Joseph et al. [34] for *C. condimenti* LMG26250T. In addition to these features, several other genomic regions and operons have been discovered that are likely to be related to the functioning of microbes in the plant environment. These include galactose/methyl galactoside ABC transporter (*galMTE* and *mglABC*) and L-rhamnose ABC transporter (*rhaTSM)*. The same genomic regions and operons were found by Grim et al. [19] in *C. muytjensii* and *C. dublinensis*. This finding can support the hypothesis that plants are the primary environmental niche for the *Cronobacter* genus bacteria, as it had been previously suggested by Iversen and Forsythe [31].

### 2.4. Genes Involved in Antimicrobial Resistance (AMR)

Antimicrobial resistance was identified by BLAST searching the sequence genome of *C. condimenti* s37 against CARD database sequence data. As shown in Table 3, the genome of *C. condimenti* s37 was found to possess 17 genes related to resistance (including, i.a., *msbA*, *marA*, *acrAB*, *kpnEFH*, *fosA8*, *adeF*, *emrR*, *rsmA*, *EF-Tu*, *glpT*, and D-alanine-D-alanine ligase gene), belonging to 11 families associated with resistance and to 13 various classes of antibiotics, including beta-lactams, tetracyclines, macrolides, phenicols, quinolones, aminoglycosides, glycopeptides, peptide antibiotics, rifamycins, nitroimidazoles, phosphonic acid derivatives, diaminopyrimidine derivatives, and elphamycins, and also genes of resistance to disinfectants and antiseptics. The low similarity of the matching region identity for some genes at the level of 30–50% is due to the limited number of sequences in the CARD database used for comparison. The nucleotide sequences of the s37 strain found in the CARD database as *vanG*, *qacJ*, and *adeF* showed a similarity of 88% to resistance genes encoding van ligase and multidrug efflux transporters in other bacteria (Blastn search analysis). Stevens et al. [35] identified 18 predicted AMR genes in all examined *C. sakazakii* strains, including 13 AMR genes, the same as in our study. One of the bacterial multidrug resistance mechanisms is the so-called efflux pump system by which the drugs are removed from the cell. Active efflux pumps enhance the virulence of many bacteria, improving their survival in hosts and allowing for cell invasion [16]. In the s37 strain, the *acrAB* operon, encoding proteins involved in this type of system, was detected [8,32]. The gene encoding for the *acrAB* operon was also reported by Jang et al. [33] in the genome of *C. sakazakii* isolated from plant-derived food products and by Chase et al. [32] in the strain *C. sakazakii* GP1999 isolated from tomato rhizoplane/rhizosphere continuum. The fosfomycin resistance gene (*fosA*) found in *C. condimenti* s37 was also reported by Jang et al. [33] in *C. sakazakii* isolates.

### 2.5. Genes Involved in Pathogenicity

The VFBD database identified 50 genes encoding virulence factors in the *C. condimenti* s37 strain, including those associated with adhesion, chemotaxis, and biofilm formation (Table S2). 

In *C. sakazakii*, the virulence genes *ompA*, *ompX*, *nanA*, *nanK*, *nanT*, *hha*, *flhA*, and *hfq* are involved in enhancing their adhesion and invasion [16,36,37]. Among the virulence-associated determinants, hemolysin type III gene (*hlyIII*), which was also reported in the genome of various strains of *Cronobacter* genus bacteria, was identified [6,38]. 

Also interesting is the presence in s37 of the genes involved in the utilization of sialic acid derivative N-acetylneuraminic acid (N-acetylneuraminic acid outer membrane channel protein NanC, nanC). Sialic acid is found in the mucin of the gut wall and the gangliosides of the brain [39]. The sialic acid utilization genes (*nanAKT*) are almost exclusively found in the *C. sakazakii* species and one branch of *C. turicensis* [36]. 

The genes *ompA*, *ompC*, *ompL*, and *ompW*, encoding porin proteins, were also identified in s37 strain. In Gram-negative bacteria, these proteins are engaged in the interaction with the cells and immune system of the host [40]. It is assumed that the outer membrane proteins OmpA and OmpX are important players in the crossing of the blood–brain barrier by *C. sakazakii* [38,41]. The literature data also indicate that the *ompW* gene contributes to *C. sakazakii* survival under conditions of osmotic and oxidative stress and that it is associated with enhanced biofilm formation [42,43]. In the genome of the strain s37, genes encoding a two-component regulatory system were also identified, encoded by the *envZ* and *ompR* genes, which are likely involved in regulating the expression of porin-encoding genes. The *fliR* and *flhA* genes, that were detected in *C. condimenti* s37, are mainly responsible for the structural elements of the flagellum. Flagella are involved in multiple mechanisms of bacterial adhesion and pathogenicity [16]. Furthermore, both *C. condimenti* strains harbor genes encoding the type IV pilus protein PilT, which is known to mediate flagellum-independent motility, referred to as twitching or gliding motility, and are associated with the virulence of various pathogens. In the genome of the strain s37, the type VI secretion system (T6SS) unique sequence such as the *vgrG* gene was also found, also acting as an effector. This system is associated with cytotoxicity and host cell invasion and allows for the pathogen to grow within macrophages [6,44]. Genes encoding identical secretion systems were detected in *Cronobacter* species [6,16]. 

Because iron is an important growth factor for bacteria, they have various mechanisms of its uptake from the environment, i.e., they produce siderophores outside the cell, which are iron-chelating compounds (e.g., enterobactin). In the s37 genome, enterobactin gene cluster (*entABCDFS*) and, additionally, siderophores of another type (*fhuABCDE*) were detected. Similar findings were reported for the majority of the *Cronobacter* strains studied by Joseph et al. [6] and for *F. helveticus* [45]. In plant growth-promoting bacteria, the siderophore enterobactin supports plant development by strengthening their resistance to pathogens [45]. *Cronobacter* species also express two ferrous transport systems, Feo (encoded by *feoABC*) and Efe (encoded by *efeOB*), which serve for Fe2+ uptake from the environment. In turn, the expression of these systems is suppressed by the interaction of iron uptake regulator proteins (Fur) binding to the appropriate promoter sites. The Fur proteins are involved in controlling intracellular iron concentration in many bacteria [9]. The presence of the *feoABC* and *efeOB* genes was also identified in the s37 strain in the present study. 

Biofilms play an important role in bacterial pathogenicity by acting as an adhesive agent, allowing for the cells to adhere to various abiotic and biotic surfaces, and also as a barrier protecting the cells from stress conditions, such as shortages of water or nutrients or the presence of antimicrobial agents. Hence, the biofilm-forming ability is a crucial trait associated with the pathogenicity of microorganisms and also with their capability to withstand food preservation and production conditions. Genes linked with the ability to form biofilms in the genome of the s37 strain, that include biosynthesis genes, like those of the colanic acid biosynthesis process (*wcaABCDEFLMK*), and those linked to the biosynthesis of flagella (*flhABCDE*, *flgA-N*, and *fliE-TZ*) were found. Genes encoding for enzymes responsible for EPS production and transport are usually clustered in large operons, like the *wca* operon, controlling colanic acid EPS biosynthesis and containing fucose and glucuronic acid [6,37]. Colanic acid is known to be one of the components of exopolysaccharides of *Cronobacter* species, the formation of which supports adhesion to the surface and increases resistance to stress factors (heat, drying, and low pH) [46]. Bacterial flagella have been shown to play an important role in biofilm formation, ensuring cell motility and surface colonization [47].

## 3. Materials and Methods

### 3.1. Materials

The study was conducted with *C. condimenti* s37, originally isolated in 2016 in Poland from commercial small radish sprouts [17]. The strain is currently stored in the collection of the Division of Milk Technology at Warsaw University of Life Sciences, Poland. The *Cronobacter* spp. strain was stored frozen on Tryptone Soy Broth (TSB) (Oxoid Argenta, Poznań, Poland) with 10% glycerol, at a temperature of −40 °C. It was recovered from the frozen state by transferring 0.1 mL of a defrosted culture into 10 mL of sterile TSB and incubation at 35 °C for 24 h. Afterwards, the strain was inoculated onto Tryptone Soy Agar (TSA) medium (Oxoid Argenta, Poznań, Poland). The motility of *C. condimenti* s37 was determined using soft-agar tubes (0.4% agar) and *E. coli* ATCC 25922 and *S. aureus* ATTC 29213 strains as positive and negative controls, respectively.

The genome of *C. condimenti* s37 was compared with that of *C. condimenti* LMG 26250^T^ and other representatives of the genus *Cronobacter* (*C. sakazakii* ATCC BAA-894, *C. malonaticus* LMG 23826^T^, *C. turicensis* LMG23827^T^, *C. muytjensii* CIP 103581, *C. dublinensis* LMG 23823^T^, and *C. universalis* NCTC 9529^T^), as well as *Franconibacter* (*F. helveticus* LMG 23733, *F. pulveris* Fp-G-601/05^T^, *F. daqui* LMG 29914^T^), and *Siccibacter* (*S. colletis* NCTC 14934^T^ and *S. turicensis* LMG 23730^T^). Characteristic features (such as source of isolation, country, phenotypic features, sequence types, and clonal complexes) of bacterial strains used in the cgMLST analysis are available in the public PubMLST database (www.pubmlst.org/cronobacter/) (accessed on 2 August 2024).

### 3.2. Draft Genome Sequence of C. condimenti s37

Genomic DNA from *C. condimenti* s37 was extracted from 500 µL of a 1-day (37 °C, 18 h) broth culture in TSB using the GenElute^TM^ Bacterial Genomic DNA kit (Sigma-Aldrich), following the manufacturer’s instructions. Sequencing was performed on the Illumina MiSeq platform (Illumina, San Diego, CA, USA), employing v3 chemistry and generating 300 bp paired-end reads with dual indexed Nextera XT libraries (Illumina). The mean insert size was approximately 250–300 bp. Quality control of the raw sequence data was performed using FastQC v0.11.8 [48]. Adapter trimming and quality filtering was performed by Trimmomatic v0.33 [49], retaining only reads with a quality score above Q30 for further analysis. De novo assembly was performed using Velvet v.1.2.10 [50], with quality assessment conducted using QUAST v4.0 [51]. The high quality of the genome assembly was confirmed via BUSCO (Benchmarking Universal Single-Copy Orthologs) analysis (v5.2.2) [52]. The DNA sequence was annotated using the NCBI Prokaryotic Genome Annotation Pipeline (v.6.6) [53] and the functional features of s37 were identified using Rapid Annotations using Subsystems Technology (RAST) [54]. 

### 3.3. Core Genome-MLST Analysis

Core genome multilocus (cgMLST) allele differences were identified using the BIGSdb MLST Genome Comparator feature of the *Cronobacter* PubMLST database (www.pubmlst.org/cronobacter/ [55], accessed on 2 August 2024). The genome of the *C. condimenti* strain LMG 26250^T^ was used as the reference genome (reference number NZ_CP012264), and the remaining representative genomes of the genus *Cronobacter* (*C. sakazakii* ATCC BAA-894, *C. malonaticus* LMG 23826T, *C. turicensis* LMG23827T, *C. muytjensii* CIP 103581, *C. dublinensis* LMG 23823T, and *C. universalis* NCTC 9529T), the genus *Franconibacter* (*F. helveticus* LMG 23733, *F. pulveris* Fp-G-601/05T, and *F. daqui* LMG 29914T), and the genus *Sicibacter* (*S. colletis* NCTC 14934T and *S. turicensis* LMG 23730T) were obtained from the PubMLST database, which was used to construct a comparative genome MLST scheme consisting of 3991 targets. cgMLST analysis and splits tree construction were performed using the genomes of the above-mentioned bacterial strains and default settings. The distance matrix was calculated in BIGSdb Genome Comparator [55]. Incomplete loci were ignored in pairwise comparisons unless the locus was missing in one isolate. Paralogous loci were excluded from the distance matrix calculation.

A description of the species, source, MLST sequence type, and PubMLST ID used in the comparative genome analysis is given in Table 4.

### 3.4. Antimicrobial Resistance Genes and Virulence Factors of C. condimenti s37 

The *C. condimenti* s37 genome sequence was analyzed for the presence of resistance and virulence genes using the Comprehensive Antibiotic Resistance Database (CARD, https://card.mcmaster.ca) and the Virulence Factor Database (VFDB, http://www.mgc.ac.cn/VFs [56], accessed on 2 August 2024), respectively.

## 4. Conclusions

The present study was focused on the draft genome of *C. condimenti* strain s37. This is the second genome sequenced strain in the species and the third reported isolate of the species. Assembly and annotation of the genome resulted in a genome of 4,590,991 bp, with a total number of genes of 4384, and a GC content of 55.7%. The genome sequence of *C. condimenti* s37 included genes responsible for heavy metal resistance, resistance to copper and potassium homeostasis, stress response associated with osmotic and oxidative stresses, resistance to antibiotics, and pathogenicity. Based on the cg-MLST analysis, a greater similarity of the s37 alleles to *S. turicensis* LMG23730^T^, *F. pulveris* Fp-G-601/05^T^, and *C. dublinensis* LMG23823^T^ was determined than to other representatives of the genus *Cronobacter*, i.e., *C. sakazakii, C. malonaticus, C. turicensis*, and *C. universalis*.

This study gives a molecular basis to understand the ability of environmental strains of *Cronobacter* to survive stressful conditions. In genome s37, genetic fragments which encode factors for enhanced environmental persistence and plant commensalism as well as numerous features that could aid in potential virulence were found. Studies on the diversity, pathogenicity, and virulence of *Cronobacter* spp. isolated from different sources are still insufficient and should certainly be continued. Especially the analysis of rare strains such as s37 is very important because it provides new information on the evolution of these bacteria. Comparative cgMLST analysis of bacteria such as s37 with other *Cronobacter* spp., as well as closely related genera *Franconibacter* and *Siccibacter*, complements the current knowledge on their adaptability to specific environments such as desiccation.

The characterization of strains isolated from foods of plant origin is very important to verify the association with cases of infections that were not caused by PIF in adults. The genetic determinants of antibiotic resistance in *Cronobacter* bacteria should be constantly investigated due to the acquisition of resistance observed in some strains and multidrug resistance observed not only in strains of clinical origin. Although *C. condimenti* is not a pathogenic member of the *Cronobacter* genus, understanding its virulence-related features may prove useful in characterizing the entire genus. 

## Figures and Tables

**Table 1:** General genome features of *C. condimenti* s37.

Feature	*C. condimenti* s37
Genome size (bp)	4,590,991
GC content (%)	55.7
Number of genes (total)	4384
Total number of RNA	96

**Table 2:** Potentially paralogous loci in the cgMLST analysis of 7 *Cronobacter* species, 3 *Franconibacter* species, and 2 *Siccibacter* species.

Locus	Product	Isolates	Isolate Count
AFK62_RS01850	efflux RND transporter permease subunit	*Cronobacter sakazakii* ^a^ *Cronobacter malonaticus* ^b^ *Cronobacter turicensis* ^c^ *Cronobacter muytjensii* ^d^ *Cronobacter dublinensis* ^e^ *Cronobacter condimenti* ^f^ *Cronobacter condimenti* s37 *Cronobacter universalis* ^g^ *Franconibacter helveticus* ^h^ *Franconibacter pulveris* *Franconibacter daqui* ^i^ *Siccibacter colletis* ^j^ *Siccibacter turicensis* ^k^	13
AFK62_RS03000	organic hydroperoxide resistance protein		
AFK62_RS05310	multidrug efflux RND transporter permease subunit AcrB		
AFK62_RS09870	general stress protein		
AFK62_RS11360	L-serine ammonia-lyase		
AFK62_RS11405	transcription antiterminator/RNA stability regulator CspE		
AFK62_RS15790	L-serine ammonia-lyase		
AFK62_RS16240	transketolase		
AFK62_RS17895	maltose/maltodextrin ABC transporter ATP-binding protein MalK		
AFK62_RS08905	PTS sugar transporter subunit IIC	*Cronobacter sakazakii* ^a^ *Cronobacter malonaticus* ^b^ *Cronobacter turicensis* ^c^ *Cronobacter muytjensii* ^d^ *Cronobacter dublinensis* ^e^ *Cronobacter condimenti* ^f^ *Cronobacter condimenti* s37 *Cronobacter universalis* ^g^ *Siccibacter colletis* ^j^ *Siccibacter turicensis* ^k^	10
AFK62_RS11020	contact-dependent growth inhibition system immunity protein	*Cronobacter dublinensis ^e^* *Cronobacter condimenti ^f^* *Cronobacter condimenti* s37	3
AFK62_RS11030	contact-dependent growth inhibition system immunity protein	*Cronobacter dublinensis* ^e^ *Cronobacter condimenti* ^f^ *Cronobacter condimenti* s37	3
AFK62_RS22640	hypothetical protein	*Cronobacter dublinensis ^e^* *Cronobacter condimenti ^f^* *Cronobacter condimenti* s37	3
AFK62_RS05535	hypothetical protein	*Cronobacter condimenti* ^f^ *Cronobacter condimenti* s37	2
AFK62_RS05550	hypothetical protein	*Cronobacter condimenti* ^f^ *Cronobacter condimenti* s37	2

^a^ *C. sakazakii* ATCC BAA-894. ^b^ *C. malonaticus* species type strain—LMG23826^T^. ^c^ *C. turicensis* species type strain—LMG23827^T^. ^d^ *C. muytjensii* species type strain—CIP103581. ^e^ *C. dublinensis* species type strain—LMG23823^T^. ^f^ *C. condimenti* species type strain—LMG26250^T^. ^g^ *C. universalis* species type strain—NCTC9529^T^. *^h^ F. helveticus* species type strain—LMG23733. *^i^ F. daqui* species type strain—LMG29914^T^. *^j^ S. colletis* species type strain—NCTC 14934^T^. *^k^ S. turicensis* species type strain—LMG23730^T.^

**Table 3:** Antimicrobial resistance genes identified in *C. condimenti* s37 using resistance gene identifier database (CARD).

Antimicrobial Resistance Gene (AMR Gene)	AMR Gene Family	Drug Class	Resistance Mechanism	Identity of Matching Region (%)	Length of Reference Sequence (%)
*vanG*	glycopeptide resistance gene cluster, D-alanine--D-alanine ligase	glycopeptide antibiotic	antibiotic target alteration	36.59	104.87
*msbA*	ATP-binding cassette (ABC) antibiotic efflux pump	nitroimidazole antibiotic	antibiotic efflux	92.27	100.00
*qacJ*	small multidrug resistance (SMR) antibiotic efflux pump	disinfecting agents and antiseptics	antibiotic efflux	45.63	101.87
*marA*	resistance-nodulation-cell division (RND) antibiotic efflux pump, General Bacterial Porin with reduced permeability to beta-lactams	fluoroquinolone antibiotic, monobactam, carbapenem, cephalosporin, glycylcycline, cephamycin, penam, tetracycline antibiotic, rifamycin antibiotic, phenicol antibiotic, penem, disinfecting agents, and antiseptics	antibiotic efflux, reduced permeability to antibiotic	89.52	97.64
*Klebsiella pneumoniae kpnF*	small multidrug resistance (SMR) antibiotic efflux pump	macrolide antibiotic, aminoglycoside antibiotic, cephalosporin, tetracycline antibiotic, peptide antibiotic, rifamycin antibiotic, disinfecting agents, and antiseptics	antibiotic efflux	73.39	100.00
*Klebsiella pneumoniae kpnE*	small multidrug resistance (SMR) antibiotic efflux pump	macrolide antibiotic, aminoglycoside antibiotic, cephalosporin, tetracycline antibiotic, peptide antibiotic, rifamycin antibiotic, disinfecting agents, and antiseptics	antibiotic efflux	73.11	99.17
*fosA8*	fosfomycin thiol transferase	phosphonic acid antibiotic	antibiotic inactivation	58.82	95.74
*H-NS*	major facilitator superfamily (MFS) antibiotic efflux pump, resistance-nodulation-cell division (RND) antibiotic efflux pump	macrolide antibiotic, fluoroquinolone antibiotic, cephalosporin, cephamycin, penam, tetracycline antibiotic	antibiotic efflux	91.91	100.00
*adeF*	resistance-nodulation-cell division (RND) antibiotic efflux pump	fluoroquinolone antibiotic, tetracycline antibiotic	antibiotic efflux	41.7	98.02
*emrR*	major facilitator superfamily (MFS) antibiotic efflux pump	fluoroquinolone antibiotic	antibiotic efflux	91.43	100.00
*Klebsiella pneumoniae kpnH*	major facilitator superfamily (MFS) antibiotic efflux pump	macrolide antibiotic, fluoroquinolone antibiotic, aminoglycoside antibiotic, carbapenem, cephalosporin, penam, peptide antibiotic, penem	antibiotic efflux	92.19	100.00
*rsmA*	resistance-nodulation-cell division (RND) antibiotic efflux pump	fluoroquinolone antibiotic, diaminopyrimidine antibiotic, phenicol antibiotic	antibiotic efflux	85.25	100.00
*CRP*	resistance-nodulation-cell division (RND) antibiotic efflux pump	macrolide antibiotic, fluoroquinolone antibiotic, penam	antibiotic efflux	99.05	100.00
*Escherichia coli EF-Tu mutants conferring resistance to Pulvomycin*	elfamycin resistant EF-Tu	elfamycin antibiotic	antibiotic target alteration	98.22	96.33
*Haemophilus influenzae PBP3 conferring resistance to beta-lactam antibiotics*	Penicillin-binding protein mutations conferring resistance to beta-lactam antibiotics	cephalosporin, cephamycin, penam	antibiotic target alteration	52.04	96.39
*Escherichia coli glpT with mutation conferring resistance to fosfomycin*	antibiotic-resistant GlpT	phosphonic acid antibiotic	antibiotic target alteration	92.43	99.56
*Escherichia coli AcrAB-TolC with MarR mutations conferring resistance to ciprofloxacin and tetracycline*	resistance-nodulation-cell division (RND) antibiotic efflux pump	fluoroquinolone antibiotic, cephalosporin, glycylcycline, penam, tetracycline antibiotic, rifamycin antibiotic, phenicol antibiotic, disinfecting agents, and antiseptics	antibiotic target alteration, antibiotic efflux	84.72	113.19

**Table 4:** List of species, sources, MLST sequence types, and PubMLST ID of strains which were used in the comparative genome analysis.

Species	Strain	Source	MLST Sequence Type	PubMLST ID
*Cronobacter sakazakii*	ATCC BAA-894	Powdered formula	1	5
*Cronobacter malonaticus*	LMG 23826^T^	Clinical	7	61
*Cronobacter turicensis*	LMG23827^T^	Clinical	19	110
*Cronobacter muytjensii*	CIP 103581	Clinical	81	598
*Cronobacter dublinensis*	LMG 23823^T^	Environmental	106	146
*Cronobacter condimenti*	LMG 26250^T^	Food	98	1554
***Cronobacter condimenti***	**s37**	**Small radish sprouts**	**98**	**1896**
*Cronobacter universalis*	NCTC 9529^T^	Water	54	84
*Franconibacter helveticus*	LMG 23733	Fruit powder	217	643
*Franconibacter pulveris*	Fp-G-601/05^T^	Fruit powder	231	611
*Franconibacter daqui*	LMG 29914^T^	Food ingredient	766	3273
*Siccibacter colletis*	NCTC 14934^T^	Food ingredient	227	1165
*Siccibacter turicensis*	LMG 23730^T^	Fruit powder	216	1179

## Data Availability

The genome of the *Cronobacter condimenti* s37 strain has been deposited in the DDBJ/ENA/GenBank databases under accession numbers RQEO01000001-RQEO01000043, with BioProject: PRJNA506216, and BioSample: SAMN10450162. It is also listed as ID number 1896 in the *Cronobacter* PubMLST database.

## Supplementary Materials

The following supporting information can be downloaded at https://www.mdpi.com/article/10.3390/ijms25168622/s1.
