# Supplementary material for: Genomic Analysis of Cronobacter condimenti s37: Identification of Resistance and Virulence Genes and Comparison with Other Cronobacter and Closely Related Species
Source: Int J Mol Sci. 2024 Aug 7;25(16):8622. doi: 10.3390/ijms25168622 (PMC11354601; doi:10.3390/ijms25168622)
Supplement: Supplementary file 1 [file ijms-25-08622-s001.zip › ijms-3111704-supplementary.pdf]

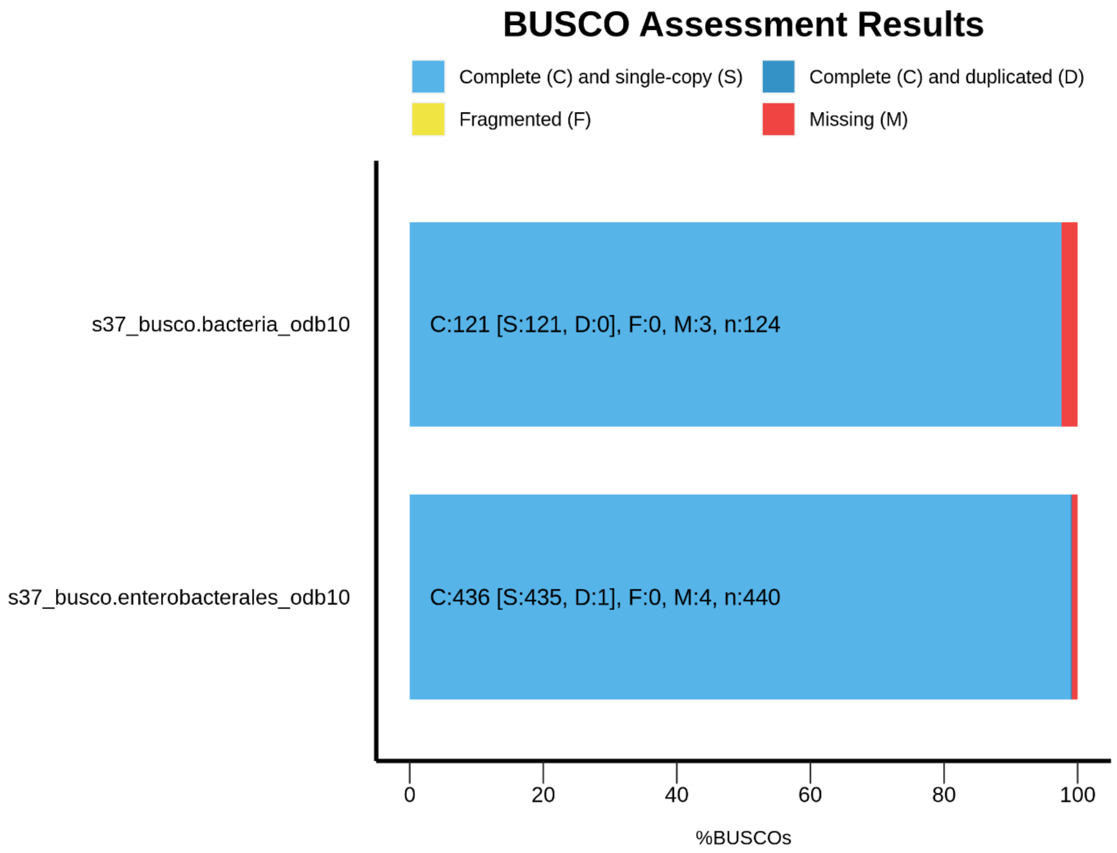

Figure S1. Completeness of the *C. condimenti* s37 genome assessed with BUSCO. The bar chart shows the percentage of missing (M), fragmented (F), complete and duplicated (C and D), and complete and single-copy (C and S) genes in the assemblies, represented by red, yellow, dark blue, and light blue bars, respectively.
