# Supplementary material for: Genomic Analysis of Cronobacter condimenti s37: Identification of Resistance and Virulence Genes and Comparison with Other Cronobacter and Closely Related Species
Source: Int J Mol Sci. 2024 Aug 7;25(16):8622. doi: 10.3390/ijms25168622 (PMC11354601; doi:10.3390/ijms25168622)
Supplement: Supplementary file 1 [file ijms-25-08622-s001.zip › Table S2.pdf]

Table S2. Genes encoding the virulence factors identified in *C. condimenti* s37 strain using VFBD database

Sequences producing significant alignments	Length (bp)	E - value	Overall identity	Gaps	Query aligned	Subject aligned
VFG000477 (rpoS) sigma S (sigma 38) factor of RNA polymerase, major sigmafactor during stationary phase [RpoS] *[Salmonella enterica* subsp. *enterica* serovar Typhimurium str. LT2]	993	0.0	88.0% (866/984)	0	3422159-3423142	989-6
VFG043545 (ECS88_3547) lipoprotein NlpI [NlpI] [*Escherichia coli* O45:K1:H7 str. S88]	885	0.0	88.6% (784/885)	0	490178-491062	1-885
VFG043544 (Z1307) outer membrane protein A [OmpA] [*Escherichia coli* O157:H7 str. EDL933]	1041	0.0	86.0% (891/1036)	3	1587877-1588912	1041-9
VFG001443 (ompA) outer membrane protein A [OmpA] [*Escherichia coli* O18:K1:H7 str. RS218]	1041	0.0	85.7% (888/1036)	3	1587877-1588912	1041-9
VFG043108 (fliG) flagellar motor switch protein G [peritrichous flagella] [*Escherichia coli* O157:H7 str. EDL933]	996	0.0	86.1% (843/979)	0	2662042-2663020	16-994
VFG043057 (fliG) flagellar motor switch protein G [peritrichous flagella] [*Salmonella enterica* subsp. *enterica* serovar Typhimurium str. LT2]	996	0.0	85.7% (837/977)	0	2662045-2663021	19-995
VFG043027 (flgG) flagellar basal body rod protein FlgG [peritrichous flagella] [*Salmonella enterica* subsp. *enterica* serovar Typhimurium str. LT2]	783	0.0	86.3% (676/783)	0	1685752-1686534	1-783
VFG043042 (cheA) chemotaxis protein CheA [peritrichous flagella] [*Salmonella enterica* subsp. *enterica* serovar Typhimurium str. LT2]	2016	0.0	83.3% (1272/1527)	0	2588571-2589738, 2590207-2590565	2001-834, 359-1
VFG043038 (cheB) chemotaxis-specific methylesterase [peritrichous flagella] [*Salmonella enterica* subsp. *enterica* serovar Typhimurium str. LT2]	1050	0.0	83.4% (875/1049)	2	2578295-2579342	1048-1
VFG035106 (ECO103_3358) hypothetical protein [SCI-I T6SS] [*Escherichia coli* O103:H2 str. 12009]	1539	0.0	83.5% (807/967)	0	3091222-3092188	1535-569
VFG043079 (flgG) flagellar basal body rod protein FlgG [peritrichous flagella] [*Escherichia coli* O157:H7 str. EDL933]	783	e-180	85.3% (668/783)	0	1685752-1686534	1-783
VFG035119 (LF82_427) hypothetical protein [SCI-I T6SS] [*Escherichia coli* O83:H1 str. LF82]	1539	e-177	83.2% (805/967)	0	3091222-3092188	1535-569

VFG035115 (EcE24377A_3124) hypothetical protein [SCI-I T6SS] [*Escherichia coli* E24377A]	1539	e-177	83.2% (805/967)	0	3091222-3092188	1535-569
VFG035103 (ECP_2801) hypothetical protein [SCI-I T6SS] [*Escherichia coli* 536]	1539	e-177	83.6% (843/1008)	0	3091222-3092188, 3092284-3092324	1535-569, 473-433
VFG035112 (ECABU_c30890) hypothetical protein [SCI-I T6SS] [*Escherichia coli* ABU 83972]	1539	e-174	83.5% (842/1008)	0	3091222-3092188, 3092284-3092324	1535-569, 473-433
VFG035102 (c3386) hypothetical protein [SCI-I T6SS] [*Escherichia coli* CFT073]	1545	e-174	83.5% (842/1008)	0	3091222-3092188, 3092284-3092324	1541-575, 479-439
VFG043063 (fliM) flagellar motor switch protein FliM [peritrichous flagella] [*Salmonella enterica* subsp. *enterica* serovar Typhimurium str. LT2]	1005	e-174	84.1% (815/969)	0	2667392-2667450, 2667480-2668389	1-59, 92-1001
VFG035117 (ECOK1_3194) hypothetical protein [SCI-I T6SS] [*Escherichia coli* O18:K1:H7 str. IHE3034]	1539	e-167	82.8% (801/967)	0	3091222-3092188	1535-569
VFG035105 (UTI89_C3191) hypothetical protein [SCI-I T6SS] [*Escherichia coli* UTI89]	1545	e-167	82.8% (801/967)	0	3091222-3092188	1541-575
VFG043049 (fliA) flagellar biosynthesis sigma factor [peritrichous flagella] [*Salmonella enterica* subsp. *enterica* serovar Typhimurium str. LT2]	720	e-165	85.4% (615/720)	0	2628628-2629347	720-1
VFG035104 (APECO1_3717) hypothetical protein [SCI-I T6SS] [*Escherichia coli* APEC O1]	1545	e-165	82.7% (800/967)	0	3091222-3092188	1541-575
VFG045800 (EC958_5032) hypothetical protein [SCI-I T6SS] [*Escherichia coli* O25b:H4-ST131]	1545	e-155	82.3% (796/967)	0	3091222-3092188	1541-575
VFG035114 (ECNA114_2855) hypothetical protein [SCI-I T6SS] [*Escherichia coli* NA114]	1539	e-155	82.3% (796/967)	0	3091222-3092188	1535-569
VFG043114 (fliM) flagellar motor switch protein FliM [peritrichous flagella] [*Escherichia coli* O157:H7 str. EDL933]	1005	e-153	83.0% (798/961)	0	2667392-2667450, 2667488-2668389	1-59, 100-1001
VFG043034 (flhA) flagellar biosynthesis protein FlhA [peritrichous flagella] [*Salmonella enterica* subsp. *enterica* serovar Typhimurium str. LT2]	2079	e-150	81.6% (1449/1776)	0	2573994-2574910, 2575102-2575960	1967-1051, 859-1
VFG043059 (fliI) flagellum-specific ATP synthase [peritrichous flagella] [*Salmonella enterica* subsp. *enterica* serovar Typhimurium str. LT2]	1371	e-136	79.9% (1021/1278)	0	2663734-2665011	16-1293

VFG043086 (flhA) flagellar biosynthesis protein FlhA [peritrichous flagella] [*Escherichia coli* O157:H7 str. EDL933]	2079	e-128	80.9% (1444/1784)	0	2574096-2574910, 2574952-2575920	1865-1051, 1009-41
VFG043094 (cheA) chemotaxis protein CheA [peritrichous flagella] [*Escherichia coli* O157:H7 str. EDL933]	1965	e-126	80.8% (1276/1580)	2	2588556-2589738, 2590170-2590565	1965-783, 396-1
VFG043099 (fliY) cystine transporter subunit [peritrichous flagella] [*Escherichia coli* O157:H7 str. EDL933]	801	e-121	84.4% (507/601)	0	2627236-2627836	698-98
VFG043110 (fliI) flagellum-specific ATP synthase [peritrichous flagella] [*Escherichia coli* O157:H7 str. EDL933]	1374	e-120	81.6% (713/874)	4	2664094-2664965	376-1247
VFG023637 (fliG) flagellar motor switch protein G [Flagella (cluster I)] [*Yersinia enterocolitica* subsp. *palearctica* 105.5R(r)]	993	e-111	82.7% (559/676)	0	2662345-2663020	316-991
VFG043101 (fliA) flagellar biosynthesis sigma factor [peritrichous flagella] [*Escherichia coli* O157:H7 str. EDL933]	720	e-106	82.2% (569/692)	0	2628656-2629347	692-1
VFG023641 (fliI) flagellum-specific ATP synthase [Flagella (cluster I)] [*Yersinia enterocolitica* subsp. *palearctica* 105.5R(r)]	1365	e-104	82.8% (599/723)	0	2663920-2664005, 2664205-2664841	202-287, 484-1120
VFG002329 (fliG) flagellar motor switch protein G [Flagella] [*Yersinia enterocolitica* subsp. *enterocolitica* 8081]	993	e-103	82.2% (556/676)	0	2662345-2663020	316-991
VFG002331 (fliI) flagellum-specific ATP synthase FliI [Flagella] [*Yersinia enterocolitica* subsp. *enterocolitica* 8081]	1332	e-103	82.5% (534/647)	0	2664205-2664851	451-1097
VFG035174 (EC55989_3335) hypothetical protein [SCI-I T6SS] [*Escherichia coli* 55989]	492	5E-94	84.7% (393/464)	0	3087025-3087488	464-1
VFG043047 (fliY) cystine transporter subunit [peritrichous flagella] [*Salmonella enterica* subsp. *enterica* serovar Typhimurium str. LT2]	801	8E-93	81.6% (550/674)	0	2627239-2627912	695-22
VFG043028 (flgH) flagellar basal body L-ring protein [peritrichous flagella] [*Salmonella enterica* subsp. *enterica* serovar Typhimurium str. LT2]	699	8E-93	83.3% (445/534)	0	1686757-1687290	166-699
VFG043096 (motA) flagellar motor protein MotA [peritrichous flagella] [*Escherichia coli* O157:H7 str. EDL933]	888	3E-92	80.7% (656/813)	2	2591572-2592383	812-1

VFG043040 (tar/cheM) methyl accepting chemotaxis protein II [peritrichous flagella] [*Salmonella enterica* subsp. *enterica* serovar Typhimurium str. LT2]	1662	5E-91	83.7% (3413/4080)	0	542465-542550, 544448-544546, 638415-638781, 2214777-2214910, 2397847-2397980, 2215054-2215186, 2580754-2581452, 2609439-2609575, 3406858-3407036, 3746663-3746799, 2802576-2802741, 1769043-1769125, 3782432-3782584, 1151732-1151829, 1769974-1770080, 638860-639087, 1768788-1768882, 2033654-2033763, 3782698-3782818, 2060501-2060553, 3371491-3371565, 3674662-3674807, 3792752-3792813, 2582206-2582285, 1746348-1746448, 3782217-3782338, 2214488-2214591, 3406609-3406679	1108-1193, 1108-1206, 848-1214, 1241-1108, 1108-1241, 964-832, 1529-831, 1241-1105, 1260-1082, 1241-1105, 1227-1062, 1111-1193, 1251-1099, 1093-1190, 1211-1105, 1293-1520, 856-950, 1214-1105, 985-865, 1111-1163, 1187-1113, 1226-1081, 1108-1169, 80-1, 1205-1105, 1466-1345, 1530-1427, 1509-1439
VFG018984 (fliI) ATPase, FliI/YscN family [Flagella (cluster I)] [Yersinia pseudotuberculosis YPIII]	1419	2E-90	82.0% (633/772)	0	2663794-2663939, 2664214-2664839	76-221, 547-1172

VFG003418 (fliI) flagellum-specific ATP synthase [Flagella (cluster I)] [*Yersinia pseudotuberculosis* IP 32953]	1401	2E-90	82.0% (633/772)	0	2663794-2663939, 2664214-2664839	76-221, 529-1154
VFG013012 (fepA) outer membrane receptor for ferric enterobactin (enterochelin) and colicins B and D [Enterobactin transport] [*Shigella flexneri* 5 str. 8401]	2241	3E-89	81.2% (1253/1544)	0	1228659-1228891, 1229077-1229341, 1229567-1229701, 1229747-1230514, 2344211-2344353	1949-1717, 1537-1273, 1038-904, 870-103, 365-507
VFG013010 (fepA) ferrienterobactin receptor precursor [Enterobactin transport] [*Shigella flexneri* 2a str. 301]	2241	3E-89	81.2% (1253/1544)	0	1228659-1228891, 1229077-1229341, 1229567-1229701, 1229747-1230514, 2344211-2344353	1949-1717, 1537-1273, 1038-904, 870-103, 365-507
VFG020224 (fliI) flagellum-specific ATP synthase [Flagella (cluster I)] [*Yersinia pseudotuberculosis* PB1/+]	1419	5E-88	81.9% (632/772)	0	2663794-2663939, 2664214-2664839	76-221, 547-1172
VFG003285 (fliI) flagellum-specific ATP synthase [Flagella (cluster I)] [*Yersinia pestis* Pestoides F]	1503	5E-88	81.9% (632/772)	0	2663794-2663939, 2664214-2664839	178-323, 631-1256
VFG003015 (fliI) flagellum-specific ATP synthase [Flagella (cluster I)] [*Yersinia pestis* Nepal516]	1521	5E-88	81.9% (632/772)	0	2663794-2663939, 2664214-2664839	178-323, 649-1274
VFG002915 (fliI) flagellum-specific ATP synthase [Flagella (cluster I)] [*Yersinia pestis* Antiqua]	1521	5E-88	81.9% (632/772)	0	2663794-2663939, 2664214-2664839	178-323, 649-1274
VFG002779 (fliI) flagellum-specific ATP synthase [Flagella (cluster I)] [*Yersinia pestis* KIM 10]	1479	5E-88	81.9% (632/772)	0	2663794-2663939, 2664214-2664839	136-281, 607-1232
VFG002660 (fliI) flagellum-specific ATP synthase [Flagella (cluster I)] [*Yersinia pestis* CO92]	1455	5E-88	81.9% (632/772)	0	-, 2664214-2664839	-, 58
